# Sensor Size Effect on Rayleigh Wave Velocity on Cementitious Surfaces

**DOI:** 10.3390/s21196483

**Published:** 2021-09-28

**Authors:** Nicolas Ospitia, Dimitrios G. Aggelis, Gerlinde Lefever

**Affiliations:** Department of Mechanics of Materials and Constructions (MeMC), Vrije Universiteit Brussel (VUB), Pleinlaan 2, 1050 Brussels, Belgium; Nicolas.Ospitia.Patino@vub.be (N.O.); Dimitrios.Aggelis@vub.be (D.G.A.)

**Keywords:** concrete, surface (Rayleigh) waves, dispersion, sensor size, aperture effect, heterogeneity

## Abstract

Concrete properties and damage conditions are widely evaluated by ultrasonics. When access is limited, the evaluation takes place from a single surface. In this case, the sensor size plays a crucial role due to the “aperture effect”. While this effect is well documented regarding the amplitude or the frequency content of the surface (or Rayleigh) wave pulses, it has not been studied in terms of the wave velocity, although the velocity value is connected to concrete stiffness, porosity, damage degree, and is even empirically used to evaluate compressive strength. In this study, numerical simulations take place where sensors of different sizes are used to measure the surface wave velocity as well as its dependence on frequency (dispersion) and sensor size, showing the strong aperture effect and suggesting rules for reliable measurements on a concrete surface. The numerical trends are also validated by experimental measurements on a cementitious material by sensors of different sizes.

## 1. Introduction

**Figure 1:**
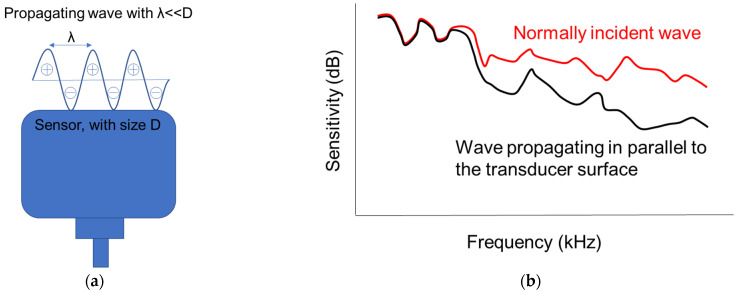
(**a**) Illustration of multiple cycles acting simultaneously on the sensor surface and (**b**) sensitivity vs. frequency curve of a transducer for waves impinging at normal angle and parallel (inspired by [9]).

Elastic wave techniques are globally used for laboratory and in-situ concrete characterization purposes, either in their passive (acoustic emission, AE) or in the active form (ultrasound). In most applications, the elastic waves are captured by piezoelectric (PZT) transducer(s) on the surface of the material. These sensors transform the pressure on their surface to an electric waveform, which is recorded and analyzed. Based on the arrival time, the amplitude and the shape of the waveform, wave speed, and attenuation can be derived, while projections to mechanical properties of concrete (Young’s and shear moduli, strength) and damage are commonly conducted with good accuracy [1,2]. Although some measurements are seemingly straightforward and can be conducted in the time domain (such as “pulse velocity” based on first threshold crossing), other more delicate parameters like the frequency-dependent velocity and attenuation curves and diffusivity heavily depend on the whole recorded waveform [3,4,5,6]. In these cases, the response of the transducers becomes crucial. The response (resonant or broadband) and the frequency band are defined by the characteristics of the piezoelectric element, such as stiffness and thickness. The response is characterized by certain calibration procedures [7,8] and is usually available for any commercial sensor for cases of vertical impinging waves as well as for waves propagating parallel to the sensor’s surface, such as guided waves in beams/plates or Rayleigh waves propagating on the surface where the sensors are attached [8,9]. In the latter case, apart from the sensitivity characteristics of the PZT element, its physical size becomes very important. The reason is simply that cycles with wavelengths shorter than the size of the element act simultaneously, with their positive and negative phases essentially cancelling each other, see Figure 1a. This is known as the “aperture effect” and has been acknowledged and studied in literature [8,9,10,11]. The consequence is that for Rayleigh or other guided waves that travel parallel to the sensor’s surface, the sensitivity to higher frequencies decreases compared to the sensitivity to bulk waves (of vertical impact to the sensor) [9,10,11,12], see also schematic representation in Figure 1b. The larger the size of the sensor, the strongest is the deviation (decrease) of sensitivity compared to normally impinging waves or to an ideal “point” receiver. Even though the decrease of sensitivity for higher frequencies due to aperture effect is well documented, there is no strong evidence in concrete literature about how the aperture influences the wave velocity measurements and more specifically the Rayleigh wave “dispersion curves” or the velocity dependence on frequency.

## 2. Wave Background

### 2.1. Rayleigh Waves and Dispersion on Concrete

**Figure 2:**
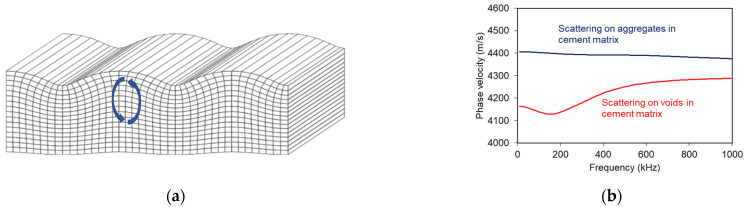
(**a**) Schematic representation of a Rayleigh wave on the surface of a medium [17] (the blue arrows stand for the ellipsoidal particle motion) and (**b**) phase velocity vs. frequency curve for the cases of stiff particles and voids in a cementitious matrix (theoretical results using the scattering model of Waterman and Truel [18]).

Rayleigh (or surface) waves are widely utilized for concrete investigation. They propagate along the surface of the material or structure, and they occupy a much higher percentage of energy than the other types of waves after a surface excitation [13]. In addition, since they propagate only in two dimensions, they do not suffer similarly much from geometric attenuation (spreading) as the three-dimensional longitudinal and shear waves [14]. This makes their detection easier than other types of waves at longer distances on concrete surfaces. Their limited penetration in terms of depth is roughly considered equal to their wavelength [15], their particle motion is elliptical, and their vertical (out-of-plane) displacement component is greater than the horizontal (Figure 2a), making their reception possible by usual PZT transducers placed on the surface [16].

Due to the microstructure of concrete including heterogeneity in ranges from μm to cm, various wavelengths can be influenced in a different way. Therefore, detailed ultrasonic studies reveal that wave propagation is dispersive or else that the wave velocity depends on the frequency. The form of dispersion (or shape of the dispersion curve) can supply information on its source (Figure 2b), such as porosity or voids [19], aggregate particles [20], damage in distributed [21,22,23] and concentrated forms [24,25,26,27], repair, and self-healing [28,29]. In addition, due to the connection of Rayleigh propagation depth to the wavelength, a layered material is by definition dispersive, which means that in such media different frequency components will propagate at different speeds. Therefore, Rayleigh dispersion curves reveal the differential stiffness of layered structures [13,23,30,31]. Additionally, the value of Rayleigh wave velocity, *C_R_,* is used for the characterization of the Young’s modulus, since *C_R_* is firmly connected to longitudinal velocity through the Poisson’s ratio [13,16]:

(1) $$C_{R}=\frac{0.87+1.12\nu }{1+\nu }\sqrt{\frac{1-2\nu }{2\left(1-\nu \right)}}C_{P},$$

where *C_P_* is the longitudinal wave velocity and *ν* is the Poisson’s ratio. Indicatively, following Equation (1), *C_R_* is approximately 55% of *C_P_*, for a Poisson’s ratio *ν* = 0.21.

Concrete strength estimations are also attempted by the Rayleigh wave velocity due to the indirect correlation of strength to stiffness [32,33,34].

In the studies where multiple wave frequencies are examined, wave velocity (of Rayleigh or longitudinal waves) shows an increase with frequency, with the strongest increasing trend at moderate frequencies (up to 100 or 150 kHz) [3,4,19,21,22,23,35]. The dispersive trend of the increase of velocity with frequency, becomes stronger with the amount of inherent heterogeneity (sand, aggregates) [4,21] and damage or air content [3,19,20,21,22]. Scattering was shown to be strongly connected to the aforementioned dispersion mainly through interaction with voids and cracks [3,19,20,21], while enhanced elastic models based on the content and size of aggregates have also been proposed [36].

Recently the influence of the sensor physical size on the Rayleigh velocity measurement in concrete has been acknowledged in [37]. There, it was shown that larger surface-bonded sensors (of 20 mm size) led to certain underestimation of Rayleigh wave velocity compared to sensors of 10 mm, using a wavelength of approximately 50 mm (the frequency was 40 kHz). This led also to a strong underestimation of the Young’s modulus by 11%. However, so far, the effect of sensor size on the frequency dependence of velocity has not been systematically studied, even though the interaction of multiple short wavelengths on the physical aperture of the sensor is bound to have an effect on the final result. This issue gains more importance as it is well known that the surface layer of concrete is normally less stiff and more porous than material deeper in the casting direction, due to settlement and bleeding, and one-sided surface wave measurements are in many cases the only way to investigate its properties.

### 2.2. Objective

In this work, a study of the aperture effect on Rayleigh wave velocity and dispersion was attempted. The main part is numerical since numerical simulation is the only way to compare the results when varying just the physical size of the sensors. In an experimental case, where sensors are compared, they may have different sizes but at the same time their overall response will be different (different PZT sensitivity curve), therefore not allowing to isolate the aperture effect. In the numerical case though, the response of the virtual receivers is flat, meaning that no bias or preference is included in the result, and the effect of the physical size can be identified. The study is conducted using surface wave propagation on the half space of a material with properties of a cementitious medium. Different frequencies are excited, and the wave is received by two sensors of three different sizes. Dispersion curves are calculated for all sensor sizes showing the noticeable “aperture” effect which becomes more evident in the case of heterogeneity, as simulated by voids in the cement matrix. An indicative set of experiments on cement mortar by using three different pairs of sensors with noticeably different size (from 4 to 41 mm) to demonstrate this effect is also presented.

## 3. Numerical Simulation and Dispersion Calculation

Numerical simulations are conducted with a commercially available software Wave2000 [38]. It computes displacement vectors by solving 2D elastic wave equations using a method of finite differences. The specific acoustic equation that is simulated is:

(2) $$\rho \frac{\theta 2u}{\theta t2}=\left(\mu +\eta \frac{\theta }{\theta t}\right)\nabla^{2}u+\left(\lambda +\mu +\varphi \frac{\theta }{\theta t}+\frac{n}{3}\frac{\theta }{\theta t}\right)\nabla \left(\nabla u\right),$$

where *u* is the displacement vector (consisting of two *u_x_* and *u_y_* components perpendicular to each other), *ρ* is the density (kg/m^3^), *λ* and *μ* are the first and second Lamé constants (Pa), *η* and *φ* are the “shear” and “bulk” viscosity (Pa·s), and *t* is time (s) [38].

**Figure 3:**
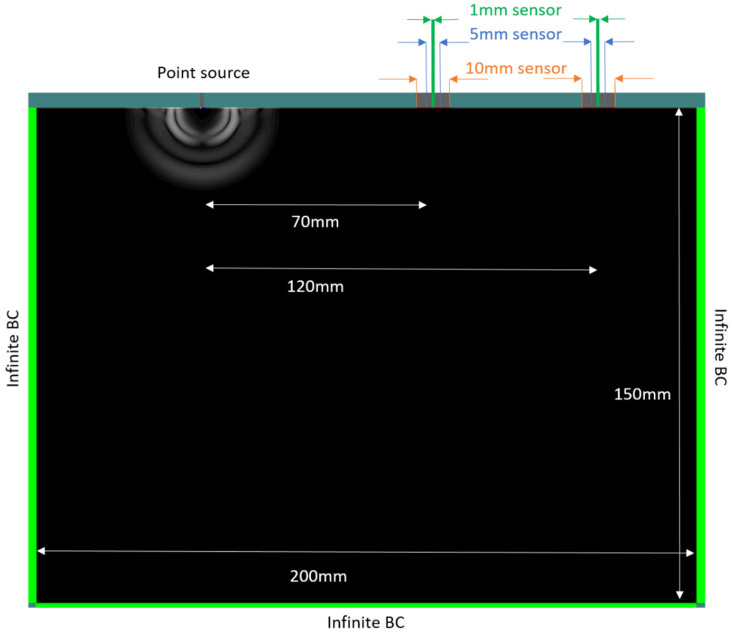
Geometry of the simulation model, showing the position of the source and sensors on the specimen.

The simulated two-dimensional geometry is given in Figure 3. The physical properties were chosen close to cementitious material: longitudinal wave velocity 4160 m/s, shear velocity 2224 m/s, and density 2170 kg/m^3^, typical of mortar. Additionally, damping attenuation was applied to the material in order to resemble the experimentally measured attenuation. This was conducted by modifying the material damping coefficients in the software until an attenuation value of 0.32 dB/mm (taken from the experiment) was reached.

Three pairs of receivers with different size were used, namely 10 (called “large” sensor), 5 (“medium” sensor), and 1 mm (“point” sensor). The receivers are set at the top right side of the geometry shown in Figure 3.

The distance from the source to the center of the closest sensor is 70 and another 50 mm to the furthest sensor. The source has a size of 1 mm. The excitation consists of a single cycle with varying frequencies, being 50, 200, and 500 kHz, applied separately. The spatial resolution of the geometry was equal to 0.1 mm and the simulation time was 100 µs.

**Figure 4:**
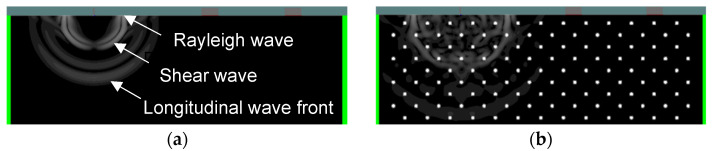
Displacement field close to the surface of (**a**) mortar without voids and (**b**) mortar with 3% voids a few µs after excitation.

The influence of heterogeneity was investigated by adding voids to the material. Specifically, Figure 4a shows the homogeneous mortar (without voids), whereas Figure 4b displays the same mortar matrix with 3% of air voids. The voids have a diameter of 2 mm, density of 1.2 kg/m^3^, and longitudinal wave velocity of 330 m/s, resembling air properties.

The calculation of the phase velocity vs. frequency curves, was conducted based on the original methodology of Sachse and Pao [39]. According to this approach, the phase of the fast Fourier transform (FFT) of the two signals (sensor 1 and 2) is calculated and unfolded for the whole frequency band. The difference of the two phases in combination with the sensor separation distance leads to the phase velocity for each frequency component. More details on the calculation are supplied in [40]. To avoid contamination of the results from components other than Rayleigh (reverberations and initial longitudinal arrivals) the signal was processed by zero-padding the rest of the waveform after maintaining the strong Rayleigh cycles, as is common in similar cases [4,41].

## 4. Results

### 4.1. Waveforms

**Figure 5:**
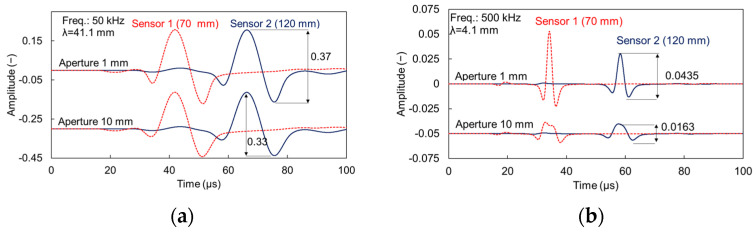
Simulated waveforms on homogeneous cement matrix for different apertures and excitation frequency of (**a**) 50 and (**b**) 500 kHz.

The description of the results starts with the pure waveforms, which are the raw data for the phase velocity calculations. Figure 5a shows the waveforms of both sensors (1 and 2 with distance 70 and 120 mm from the excitation, respectively) for the cases of 1 and 10 mm aperture and the 50 kHz excitation on plain material. Visual comparison shows no strong differences in shape or amplitude between the waveforms captured by the different apertures. The waveforms of sensor 1 start with a weak arrival at approximately 19 μs, while the strong Rayleigh contribution follows between 30 and 60 μs. Figure 5b exhibits the corresponding waveforms from the excitation of 500 kHz. In this case, the waves are much “sharper” because of the shorter period. Additionally, a change of shape starts being evident with the wave captured by the large sensor being “stretched” in time domain, while the amplitude is certainly decreased, close to one third of the point sensor (indicatively 0.0163 compared to 0.0435 for 120 mm of propagation). These are already strong indications of the aperture effect that are evident for the higher frequency pulse even from time domain and in homogeneous medium.

**Figure 6:**
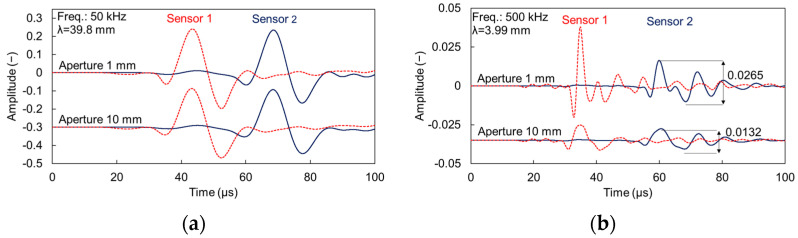
Simulated waveforms on cement matrix with 3% voids of 2 mm size for different apertures and excitation frequency of (**a**) 50 and (**b**) 500 kHz.

Moving to the material with voids, the waveforms of low-frequency excitation (50 kHz) are shown in Figure 6a. The waves do not show strong discrepancies compared to the homogeneous case. This is attributed to the high ratio of wavelength over diameter, which for this case is approximately 20 (λ ≈ 40 mm, void diameter 2 mm). The situation changes when looking at the highest excitation frequency used (500 kHz, Figure 6b). In this case the existence of more cycles is evident, indicating the effect of scattering on the voids. Considering the nominal excitation frequency, the wavelength is approximately 4 mm, being already of the order of the void diameter.

Apart from the general shape and amplitude observation, it is noteworthy that the large sensor consistently leads to underestimation of the Rayleigh velocity. This is typically measured by the delay of a characteristic point of the Rayleigh portion (e.g., the maximum or minimum point of the large cycle) between the two sensors. For the 500 kHz excitation of the reference mortar (waveforms in Figure 5b) the Rayleigh velocity is calculated at 2079 m/s for the 1 mm sensor, while at 2006 m/s for the 10 mm sensor. Concerning the heterogeneous case with voids (Figure 6b) the corresponding values are 1993 m/s for the 1-mm sensor and 1926 m/s for 10 mm. In both cases the velocity decrease is approximately 3.5%, owing only to the larger sensor size. This effect is negligible for the 50 kHz excitation with differences between apertures limited to 5 m/s (or approximately 0.2%) either for reference or heterogeneous mortar.

### 4.2. Dispersion Results

**Figure 7:**
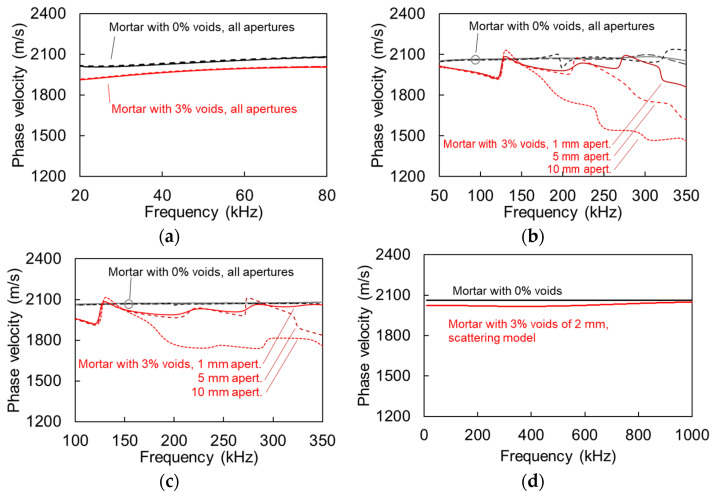
Rayleigh dispersion curves for mortar with 0% and 3% voids and excitation frequency of (**a**) 50, (**b**) 200, (**c**) 500 kHz, and (**d**) theoretical dispersion curve through scattering model.

Figure 7a shows the phase velocity vs. frequency curve for the case of plain mortar and for the mortar with 3% of voids for the 50 kHz of excitation. Results from all apertures are included for both materials, but as the curves are almost identical, they are not separately addressed in the legend for simplicity. There is a small but clear difference of approximately 60 m/s between the 0% and 3% void curves, owing to the decrease of the effective elastic modulus of the material. For the excitation frequency of 200 kHz, the dispersion curves are shown in Figure 7b. For homogeneous material, the different apertures do not lead to systematic differences and therefore, are not separately identified for simplicity. The curves for the heterogeneous media, however, exhibit distinct characteristics. First, all are less smooth than the homogeneous, exhibiting small local peaks and troughs, which are typical of heterogeneous media [3,21,22,23]. The average level of the 1 mm aperture curve is approximately 60 m/s below the plain material for the band between 100 kHz and 300 kHz. Interestingly, the aperture of 5 mm is in very good agreement with the 1 mm curve for the lower band of frequencies and starts to deviate above 240 kHz. The dispersion curve of 10 mm aperture is also in good agreement for low frequencies but starts to deviate as of 160 kHz. Therefore, it is seen that for low frequencies the dispersion curves are repeatable, when the aperture effect is negligible. However, when frequency increases and the wavelength becomes small enough to be comparable to the sensor size, the curve starts deviating from the “ideal” one. Indeed, the frequency of 240 kHz where the curve of the 5 mm sensor starts to deviate corresponds to the 8.5-mm wavelength. For the 10-mm sensor, the deviation starts at 160 kHz with a corresponding wavelength of 13 mm.

Concerning the higher frequency of excitation (nominally 500 kHz) results are depicted in Figure 7c. The 1 mm curve is almost identical to the 200 kHz case. Differences are noticed for the 5 mm aperture curve, as it starts to deviate from 310 kHz (λ ≈ 6.5 mm), while the 10 mm aperture curve deviates again as of 160 kHz (λ ≈ 13 mm). It is noticed that the dispersion results from different sensors are in good agreement for low frequencies. The aperture effect starts being evident resulting in lower phase velocity for wavelengths reaching the order of the transducer size D, and specifically when λ < 1.3–1.5 D.

## 5. Discussion

Figure 7d shows the calculated dispersion curves for the two cases (homogeneous matrix and matrix with 3% of voids of 2 mm) based on the scattering model of Waterman and Truel [18]. This concerns elastic scattering only; therefore, damping is not included. The original output is the longitudinal wave velocity. The Rayleigh wave velocity, C_R_, presented in Figure 7d was consequently calculated according to Equation (1). The Poisson’s ratio was considered 0.25 without variation with frequency. While this is an assumption, it is not far from reality, since the “dynamic” Poisson’s ratio of cementitious media, measured by ultrasound is just slightly higher than the one measured in quasi-static loading [42]. The curve of material with voids is theoretically predicted at 50 m/s lower than the plain mortar for the frequency band of interest (below 300 kHz), while at higher frequencies the velocities converge to the value of the matrix as is typical for scattering media.

The above analysis shows that with a small aperture sensor, wave velocity results fall very close to the theoretically expected ones for homogeneous as well as heterogeneous medium with small content of voids or pores. However, when the aperture, D, is comparable to the wavelength (λ ≤ 1.3D) or smaller results strongly deviate, underestimating the wave velocity by as much as 15%. As an example, looking at Figure 7c, for the heterogeneous case of 3% voids, the 1-mm sensor results in a phase velocity of 2005 m/s at 250 kHz, with the theoretical value calculated by the scattering model being 2018 m/s, showing close agreement. However, the 10 mm aperture shows 1762 m/s, which is a deviation of 13%.

**Figure 8:**
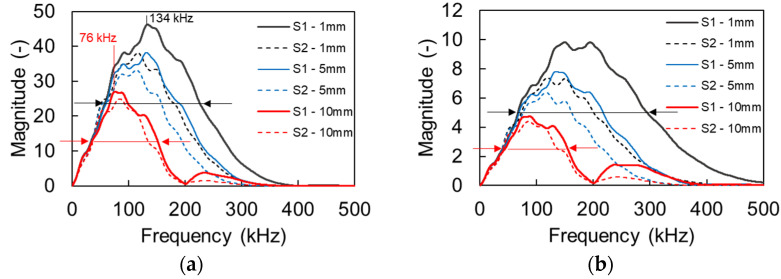
FFT magnitude of different aperture sensors for excitation frequency of (**a**) 200 and (**b**) 500 kHz.

Figure 8 shows the FFT of the responses for S1 and S2 for 200 kHz excitation (a) and for 500 kHz (b) for all apertures. Due to the imposed damping, the main content is clearly downshifted, as also experimentally observed in cementitious media [20]. For the case of 200 kHz, the peak frequency of the first sensor of 1 mm is at 134 kHz (propagation of 70 mm). For the same distance from the source the large sensor of 10 mm, exhibits a peak frequency of 76 kHz evidently showing the aperture effect. The effect is also similar in Figure 8b for the excitation of 500 kHz with a strong downshift of the peak frequency as the aperture increases from 1 to 10 mm. The horizontal lines with the arrows in Figure 8 indicate the bandwidth with half the peak magnitude for the 1 mm (black) and the 10-mm (red) sensor size, evidently showing once again the strong aperture effect, since most of the higher frequency content is eliminated for the large aperture. In Figure 8b the half peak content of the 10 mm is limited to 155 kHz, while for the same excitation, medium, and distance it expands up to 300 kHz for the 1-mm aperture. Essentially, the content above 150 kHz is canceled out in the large sensor, while it survives in the point sensor. This strong downshift is also the reason that the numerical dispersion curves in Figure 7 are presented up to 350 kHz, as the content for higher frequency bands is severely compromised or eliminated.

According to the presented results, the wave velocity is reliably calculated when the wavelength is much longer (+50% or more) than the aperture of the sensor. In cases where the wavelength drops to physical lengths similar to or lower than the physical aperture of the sensor, the velocity calculated by waveforms received on the surface is lower than the corresponding of an “ideal” point sensor, or the one predicted theoretically. This reduction can be even of the order of 15%, an artifact that has not been clearly recognized in literature and that can lead to strongly misleading results concerning the E-modulus and damage content. This becomes especially important when results need to be compared to theoretical dispersion curves (scattering, enhanced models), where for high frequencies the curves exhibit a deviation, owing only to the influence of the sensor size and not to material properties or heterogeneity.

## 6. Experimental Part

**Figure 9:**
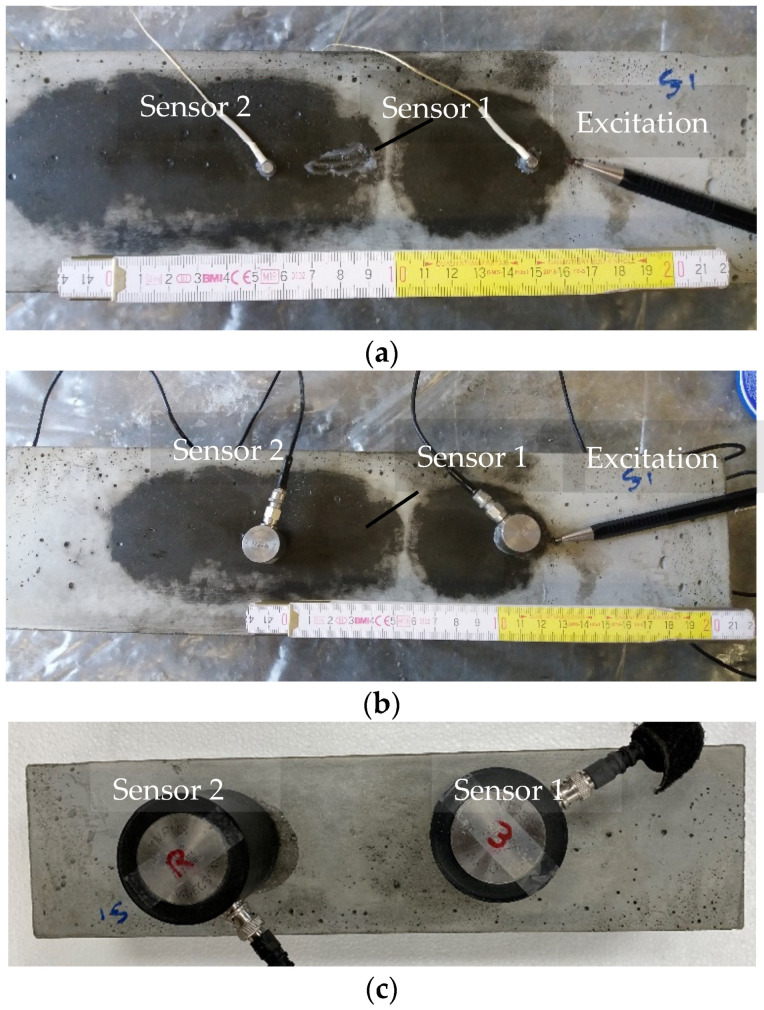
Photographs of surface wave measurements on concrete using different sensors: (**a**) Mistras pico sensor with 4-mm size, (**b**) Mistras R15 with 17.35-mm size, and (**c**) Olympus videoscan with 41.5-mm size.

For the experimental verification, different piezoelectric sensors with large variation in size were selected in order to highlight the aperture effect. Namely, these were Olympus videoscan V1012 with a central frequency of 250 kHz and diameter of contact surface of 41.5 mm, Mistras Group R15 150 kHz resonant sensor with size 17.35 mm and the Mistras Group Pico sensor (with a sensitivity peak at 450 kHz but more broadband than R15) with size of 4 mm. A thin layer of roller bearing grease was applied between the sensors and the surface of the specimen to enhance acoustic coupling. In all cases, no external pressure was applied on the sensors. Measurements were conducted on the bottom surface of the specimens (considering casting direction) which was smooth due to the contact with the steel mold and no extra preparation was necessary. The signals (waveforms) received by the sensors were pre-amplified by 40 dB and were acquired through a Micro II Digital AE Data Acquisition System from Mistras Group (Princeton Junction, NJ, USA) with a sampling rate of 10 MHz. Ultrasonic measurements were made on the surface of a mortar specimen of 400 × 100 × 100 mm. For completeness, the w/c ratio was 0.35, the maximum aggregate size was 0.85 mm, and the sand/cement ratio was 2. The sensors were placed along the central longitudinal axis of the specimen and in order to increase the representativity, the distance between them was changed from 50 to 120 mm to account for different parts of the specimen. The Rayleigh dispersion curve was derived from the waveforms of the two receivers after a broadband excitation of a mechanical pencil lead break (HB 0.5 and length 4 mm) 10 mm away from the first sensor. Figure 9 shows photographs of typical tests. More than ten individual tests were conducted with each type of sensors to evaluate the experimental scatter. It is mentioned that excitation is possible with different transducers and driving frequencies. In this study, pencil lead excitations were chosen because they are broadband and repeatable and are casually used as reference sources [20,43], allowing an identical source for all the different receivers. This way, any changes in the waveforms and eventually in the dispersion curves can be safely attributed to the receiver’s behavior (sensitivity and aperture).

**Figure 10:**
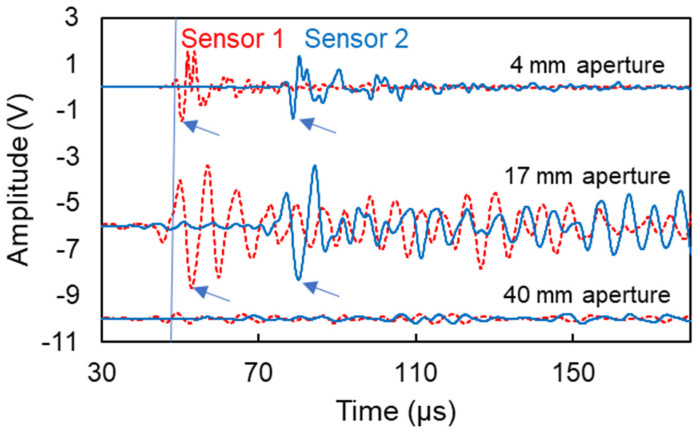
Typical surface waveforms collected by two sensors for different apertures. The arrows identify characteristic Rayleigh points used for velocity calculations. The vertical axis has been offset for the different apertures for clarity, while the amplitude of the waveforms has not been altered.

Figure 10 shows typical waveforms from all three pairs of transducers. The channels were synchronized, meaning that when a “threshold crossing” was marked in one channel (shown by the vertical line at 50 μs), recording started at both channels (sensors 1 and 2). The strong Rayleigh cycle is present and characteristic points that are used for velocity calculation can be clearly identified in the waveforms of the small sensors of 4 mm, see arrows in Figure 10. The 17-mm aperture also shows a strong Rayleigh portion, although the ringing of the strongly resonant sensor is obvious by the additional cycles. In both of these cases, the waveforms were clear enough to allow treatment towards phase velocity calculations. For the case of the large sensors of approximately 40 mm size, the signal is compromised, and the Rayleigh portion is not evident. This is expected due to the aperture effect and the aforementioned elimination of the content of the successive cycles acting upon the surface of the sensor.

Based on a characteristic Rayleigh point (e.g., negative peak as indicated by the arrows in Figure 10), the Rayleigh wave velocity is calculated at 2402 m/s using the small sensors and 2173 m/s using the medium sensors, while it was not straightforward to identify such points for the large sensors.

**Figure 11:**
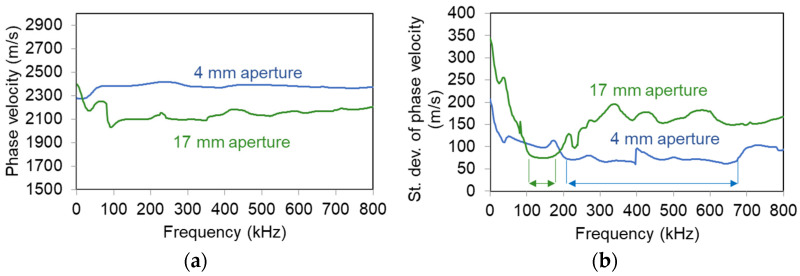
(**a**) Rayleigh dispersion curves on concrete surface with sensors of different apertures and (**b**) standard deviation of Rayleigh dispersion curves. The arrows indicate the band of lower standard deviation.

Figure 11a shows the experimental Rayleigh dispersion curves directly from the surface measurements for the small and medium sensors. It is evident that the curves derived from the medium sensors exhibit a significant decrease of phase velocity compared to the small ones. The difference is approximately 250 m/s in absolute value for most of the frequencies, representing a change of 12%. The aforementioned curves are averaged after several individual tests, by applying multiple pencil lead break excitations. To examine the repeatability of the results, the standard deviation of different curves is shown in Figure 11b. There, one can see that the R15 sensors exhibit their lowest standard deviation (70 m/s) around 150 kHz, something compatible with their sensitivity, implying that the results between 100 to 200 kHz are more reliable. Pico sensors show a standard deviation of approximately 65 m/s for the band between 200 and 700 kHz being again in accordance with their sensitivity curve and showing a much broader band of higher reliability. These areas of higher reliability are indicated by horizontal arrows in Figure 11b.

## 7. Conclusions

The present study clarifies the effect of sensor size, not only on the content of surface waves but also on the measured Rayleigh wave velocity. The importance lies in the fact that in surface ultrasonic measurements on cementitious mixtures the wave propagates in parallel to the sensor and multiple wavelengths destructively interfere. Practically, it was seen that:

- The Rayleigh pulse velocity as measured from time domain is underestimated by 3.5% for large sensors (wavelength smaller than 1.5 times the sensor size). Experimentally, this difference reached even higher values above 10%;
- The Rayleigh dispersion curve is strongly influenced by the sensor size. Specifically, the velocity starts to deviate from the point the wavelength becomes about 1.3 to 1.5 times the sensor size or lower;
- Up to that point (roughly wavelength 1.5 times the sensor size) there is no strong influence of the sensor size. Practically, for concrete media, a sensor of 4 cm diameter would lead to reliable Rayleigh wave velocity measurements up to approximately 35–40 kHz, while a sensor of 1 cm diameter, would be reliable up to 160 kHz. For higher frequencies, the velocity curve vs. frequency could be underestimated by up to 15%.

This study has implications for assessment of material in the lab and in situ. Wave velocity is empirically correlated with the strength of concrete, while it is used for assessment of stiffness and voids. Concrete is heterogeneous and experimental scatter in all measured properties (as well as ultrasonic velocity) is expected. A deviation of 15% on top of that would definitely lead to erroneous conclusions about the quality or damage content.

## Data Availability

The data of this study are available upon request, where justified, by email to the corresponding author.
